# Low carbohydrate diets in family practice: what can we learn from an internet-based support group

**DOI:** 10.1186/1475-2891-5-26

**Published:** 2006-10-02

**Authors:** Richard D Feinman, Mary C Vernon, Eric C Westman

**Affiliations:** 1 Department of Biochemistry, State University of New York Downstate Medical Center, Brooklyn, NY 11203, USA; 2 Private Bariatric and Family Practice, and Clinical Faculty, University of Kansas School of Medicine, Lawrence, KS, USA; 3 Department of Medicine, Duke University Medical Center, Durham, NC, USA

## Abstract

The Active Low-Carber Forums (ALCF) is an on-line support group started in 2000 which currently has more than 86,000 members. Data collected from posts to the forum and from an on-line survey were used to determine the behavior and attitudes of people on low carbohydrate diets. Members were asked to complete a voluntary 27-item questionnaire over the internet. Our major findings are as follows: survey respondents, like the membership at large, were mostly women and mostly significantly overweight, a significant number intending to and, in many cases, succeeding at losing more than 100 lbs. The great majority of members of ALCF identify themselves as following the Atkins diet or some variation of it. Although individual posts on the forum and in the narrative part of our survey are critical of professional help, we found that more than half of respondents saw a physician before or during dieting and, of those who did, about half received support from the physician. Another 28 % found the physician initially neutral but supportive after positive results were produced. Using the same criteria as the National Weight Registry (without follow-up) – 30 lbs or more lost and maintained for more than one year – it was found that more than 1400 people had successfully used low carb methods. In terms of food consumed, the perception of more than half of respondents were that they ate less than before the diet and whereas high protein, high fat sources replaced carbohydrate to some extent, the major change indicated by survey-takers is a large increase in green vegetables and a large decrease in fruit intake. Government or health agencies were not sources of information for dieters in this group and a collection of narrative comments indicates a high level of satisfaction, indeed enthusiasm for low carbohydrate dieting.

The results provide both a tabulation of the perceived behavior of a significant number of dieters using low carbohydrate strategies as well as a collection of narratives that provide a human perspective on what it is like to be on such a diet. An important conclusion for the family physician is that it becomes possible to identify a diet that is used by many people where the primary principle is replacement of starch and sugar-containing foods with non-starchy vegetables, with little addition of fat or protein. Used by many people who identify themselves as being on the Atkins diet, such a strategy provides the advantages of carbohydrate-restricted diets but is less iconoclastic than the popular perception and therefore more acceptable to traditional nutritionists. It is reasonable for family practitioners to turn this observation into a recommendation for patients for weight control and other health problems.

## Background

Strategies for weight loss and control of diabetes and cardiovascular disease based on carbohydrate restriction continue to be controversial. Whereas the obesity epidemic is prima facie evidence for the limitations of traditional approaches and published studies continue to bring out the efficacy and safety of low carbohydrate diets [1-7], official agencies and the media offer little support for the family physician and the individual patient considering such a diet [8]. A major problem, in our view, is that the most popular of reduced carbohydrate approaches, the Atkins diet, is an ad lib diet with recommendations only to minimize carbohydrate intake [9]. As a result, little is known about what dieters actually do, and workers in nutrition have consistently assumed that the lack of proscription against fat and protein means that this constitutes a specific recommendation to increase consumption of these macronutrients. More generally, we would suggest that the nutritional literature is lacking in what might be called a human perspective, that is, relevant information that is lost in the formalism of medical reporting.

This communication describes information from an on-line support group, the "Active Low-Carber Forums (ALCF)" [10], about the behavior of dieters on low carbohydrate diets. The use of an online site, while it falls into the category of self-reporting, has several advantages and unique characteristics.

First, the site is primarily a support group, that is, members join the group in order to share experiences and, because the group is anonymous and outside a clinical setting, have little need to satisfy a mentor or personal physician and thereby less obvious cause for bias in reporting.

In addition, the requirement for joining the group includes listing weight data and information on diet plan used. Thus, a degree of effort is required of those people who will be counted in the study and one can assume a certain level of seriousness. The personal and emotional element that bears on compliance and that is necessarily lost in statistics is salient in the forum if not always easy to quantify. It is important to emphasize that whereas bias may appear in any human report, in many cases, perceptions may be as important as established facts and the survey may be one of the most informative avenues to determine this factor.

Here we describe results of examination of ALCF emphasizing an online survey.

## Methods

Data for the study came from narrative information on the ALCF website posted by members, and primarily from a survey posted on the ALCF website.

### Survey

The online survey was based on the Unit Command Climate Assessment and Survey System (UCCASS) (pronounced yoo-kas) and implemented by the director of the forum, Wa'il al Wohaibi. UCCASS is a web-based survey script written in PHP for online surveys and questionnaires. Details and documentation are available at the UCCASS website [11].

The survey is available only to members of the low carbers forum at the website [10]

The completed survey is shown in the appendix [see Additional File 1] and can be seen (also requiring membership) in its original format at the Forums website [12].

Instructions to the survey:

The purpose of the study is to determine the eating patterns, attitudes and general dieting habits of members of the forum as an example of a group following a low carb lifestyle.

Carbohydrate restriction continues to be of importance as a method for weight reduction and treatment for diseases such as diabetes and cardiovascular disease. Scientific studies, however, are largely restricted to an abstract, experimental setting and there is a lack of information as to what people really do on low carbohydrate diets and how they feel about them. This survey is designed to help provide this information. The purpose is neither to support nor to criticize any diet but only to provide information.

Confidentiality: all information is strictly confidential and will be reported as group data unless individual permission is obtained in advance. In the final publication, posts on the forum may be presented. We will not use these without members' prior permission and no identifying ID will be used.

There are 27 questions in this survey. With subsections, there are a total of 59 multiple choices. The survey will take 5–10 minutes.

**Click on the link below **to start taking the survey. Please make sure to fill out the survey carefully, and answer as many questions as possible. **Once the survey is answered, it cannot be re-taken or changed.**

### Survey groups

Because the rationale of the survey is that the group was self-selected before the survey, that is, less influenced by the formal experimental nature of the questionnaire, we originally set a cut-off date (August 17, 2005, 28 days from first posting). Respondents who registered after the cut-off were tabulated separately from those who had already been members on the cut-off date. Results for these two groups were tabulated separately and, as noted, below, little difference was actually found between the two groups. In the results, dates are given for data values where they differ between groups. Also, the survey is still active and here, again, there is little change in the percentages of answers with time.

### Filters

Correlations were obtained by use of a filter procedure in the UCASS software. With this procedure it is possible to filter the results of the entire survey based on answers to specific questions. For example, the responses of the sub-group of responders who lost more than 30 lbs could be separately tabulated and compared with the group at large or other sub-groups whose answers had been filtered. As implemented the software has a privacy protection feature that prevents narrow filters to be used for identifying individual responders. The default setting of 3 was used, that is, if 3 or fewer surveys match the filter criteria, the results cannot be seen. This is to maintain anonymity in answering the questionnaire.

### Internal controls

Because filling out an online survey has no controls for attention of respondents and because there are unknown human variables, in order to get some idea of the reliability of answers, a few controls were built in by asking questions in different ways in different places in the questionnaire. These are discussed in the results but, for example, we asked in **Question 2**. "Have you kept at least 30 lbs off for one year or more?" and then again in **Question 38**. "Were you able to maintain at least 30 lbs of the weight you did lose for a year or more?" Variations in these answers gave us a rough measure of reliability which was typically greater than 90 %.

### Background and activity of ALCF

ALCF was started in 2000 by the current director, Wa'il al Wohaibi. The forum accepts members who are asked to enter the following information:

Start weight: Your starting weight in pounds (before you started your diet) in pounds. (Required)

Current weight: Your current weight, today, in pounds. (Required)

Goal weight: Your goal weight in pounds. (Required)

Height: Your height, please indicate units (inches or centimeters) (Required)

Gender; Male or Female? (Required)

LC Since; When did you start low-carbing? (Required)

LC Plan; Which low-carb plan do you follow? (Required)

LC Books you have read; List some of the low-carb books you have read, this will help users discuss books they are familiar with.

(Required)

ALCF as of May 28, 2006 has 86, 376 members and the site notes that "**1,185,766 **lbs lost by **57,654 **members"

## Results and discussion

Performance on the survey as of September 18, 2005 are shown in Appendix 1 (Additional Files 1) and the most current results are available on the internet at the website [12].

As noted in Methods, the original design of the survey was to run for one month.

Number of members who took the survey before the cut-off: 2, 319

Total number of respondents who registered and took the survey until January 24, 2006: 3,134

### Members and respondents

**Figure 1:**
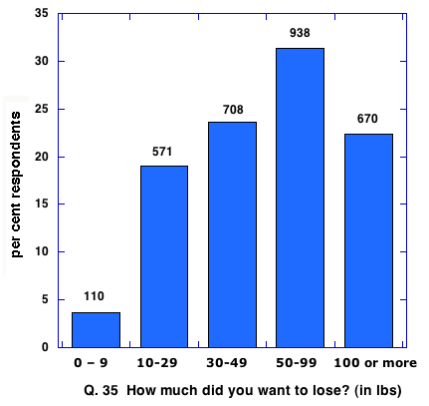
Intended weight loss of respondents to questionnaire.

The membership of ALCF is currently 83 % women, which is reflected in the makeup of respondents to the questionnaire (as of January 24, 2006, 2579 or 82.3 % women). The age distribution (from **Q. 20**) showed 61 % of respondents between 30 and 49 years of age. We did not request physical data on the questionnaire but asked for goals in weight loss in **Q. 35**. The responses indicate that the starting weights must have been very high with more than half of the people surveyed indicating that they had wanted to lose more than 50 lbs and 22 % intending to lose 100 lbs or more (Figure 1). In summary, the survey population was largely middle aged women whose goal was to lose a large amount of body mass.

#### Diet plans

A non-systematic scanning of posts on the forum suggested that most members used a personal variation of a published diet. When we asked this question specifically (**Q. 35**.), however, we were surprised to find that 55 % identified themselves as following the Atkins diet and another 19 % as following "My own variation of Atkins." When results were filtered to specifically look at the group who had lost 30 lbs or more and kept it off for one year or more (30+1 yr) we found similar results (58 % Atkins and 22 % variation of Atkins).

This is significant in that although there are many low carbohydrate strategies available to patients, the Atkins diet is taken as synonymous with all low carb strategies even though from previously published reports, anecdotal evidence and the survey presented here, there is great variation in what patients actually consume.

The meaning for the family practitioner is that the name "Atkins diet" appears to be a permanent fixture. However, outside of the proviso on carbohydrate reduction, it is quite flexible and individual practitioners can guide patients or design individual plans. For example, insofar as the Forum is generalizable a family practitioner can recommend a diet that replaces starch with non-starchy vegetables as a general strategy. This approach is perceived as the major change by a successful group of dieters and would hardly be criticized by most nutritionists.

### The 30 lb benchmark

The survey was primarily intended to assess eating patterns and the perceptions of dieters. We sought only a rough measure of actual weight loss. For this, an arbitrary point of 30 lbs was set as a rough indicator of the success of dieting (**Q. 1**) and 62 % of respondents indicated meeting this mark. We also asked whether this weight loss had been maintained for one year (**Q. 2**). This was done with reference to the National Weight Registry (NWR) cutoff that has set a standard of having lost 30 lbs or more and kept it off for one year as a benchmark for successful weight loss (see e.g. [13]). Although the original intent of NWR was similar to our own – to determine behavior of dieters – it is widely quoted that their identification of 4, 000 participants over an approximately 10 year span, most of whom had been on a low fat diet, is proof for the efficacy of such a diet. By comparison, on the one month cut-off, we had identified 1, 088 dieters using low carbohydrate diets who had met the NWR criteria. As of January 24, 2006, the number was 1423 suggesting that whatever other information comes out of the NWR study, evidence for superiority of low fat approaches is not a reasonable conclusion. Most recently, the NWR has reported an increase in the daily percentage of calories from fat and the total amount of saturated fat from 1995 to 2003 while carbohydrate decreased from 56.0% to 49.3% in this period [14]. In addition, the limited population covered by the NWR is indicated by the fact that 87 % of respondents to the questionnaire had never heard of the registry and 18 respondents had met their criteria, tried to register but never heard from them.

### Reliability of 30 lb weight loss for one year

The NWR found that for those patients who had medical records, the reliability of recollection was high and results generally did not require medical validation. Because the ALCF is voluntary and members are motivated to join to share successful experiences rather than being rewarded for success by experimenters, we think the reported values have substantial validity. The data can, of course, be taken, simply as perceptions of people who thought they had lost 30 lb and kept it off for one year. In any case, as a means of built in control (see Methods) we checked responses within the questionnaire. We filtered the results for those who answered Yes to **Question 2**. "Have you kept at least 30 lbs off for one year or more?" This subset was examined for their answer to control questions. **Question 1**:"Have you lost 30 lbs (or more) on a low-carb plan?" This should have given 100 % yes but was found to be only 95.78 %. Similarly, on **Question 38**: "Were you able to maintain at least 30 lbs of the weight you did lose for a year or more?" 96.81 % of respondents replied yes. In other words, about 50 people in the survey were confused about the question, were not paying attention or were otherwise unreliable.

### Lipid profile

There have been several reports on the effects of low carbohydrate diets on lipid profiles either alone in comparison to low fat diets (Reviews: [4,15]). The general picture that emerges is that carbohydrate restriction leads to a marked reduction in triglycerides (TAG) – this is one of the most reliable features of any dietary intervention – and improvement in HDL. Changes in total cholesterol and LDL tend to be variable on low carbohydrate diets but are generally considered to go down if there is weight loss. Of current interest is the report by Krauss, et al. [16,17] that if macronutrient composition and caloric restriction are changed sequentially, most of the beneficial effects in a low carbohydrate diet occurs during the (eucaloric) change in macronutrients whereas the beneficial effects in a low fat diet require weight loss. These results confirm the original report by Sharman, et al. that benefit of a low carb diet does not require weight loss [18] and highlight the limitations of low fat diets where improvement in lipid markers is more dependent on successful weight loss.

The survey asked if participants had had blood lipids measured before and after going on a low carb diet (**Q. 21**). Forty per cent of the total group and 51 % of the 30 lb+1 yr group had done so. As expected, from these generalizations, most of responders to the survey who had lipids measured (**Q. 22.-25.**) reported a decrease in TAG (68 %) and an increase in HDL (49 %) (Table 1). The group that had kept 30 lb off for a year did noticeably better than the group as a whole on these markers (76 % and 55 %, respectively). The table shows that 60 % of the total group and 65 % of the 30 lb+1 yr group claimed lower total cholesterol and lower LDL which was greater than the number who had improved values for HDL. Based on previous studies in the literature, one would have to consider the value for triglycerides as low. It would be expected that almost everybody in the 30 lb+1 yr group would have had decreased triglycerides. We think that these data can only be taken as semi-quantitative and it is unlikely that respondents actually went back and checked medical records.

**Table 1:** Effect of dieting on lipid profile

**Marker**	**Group**	**increased (%)**	**no change (%)**	**decreased (%)**
**Total cholesterol**	all	11	27	**62**
	30 lb+1 yr	10	21	**68**
**LDL**	all	12	30	**60**
	30 lb+1 yr	10	25	**65**
**HDL**	all	**49**	31	21
	30 lb+1 yr	**55**	25	20
**Triglycerides**	all	4	27	**68**
	30 lb+1 yr	3	21	**76**

Responses to questions 22.-25. from survey. Improvements shown in bold

### Physicians responses and interactions

Approximately half of the responders to the survey said that they had consulted a physician before or during their diet (**Q. 32.**). One of the encouraging results from the survey was that, when queried as to how they would describe support they received (**Q. 32.**), 990 (56%) of the entire group and 507 (55 %) of the 30+1 yr group who had consulted a physician reported that the physician or health professional was supportive. An additional 28 % and 32 % of the total and 30+1 yr groups reported that the physician "did not have an opinion but was encouraging after seeing results." Only 6 % of responders indicated that "they were discouraging even after I showed good results," which may be a surprising result depending on one's relative expectations of evidence-based medicine vs. prejudice against the Atkins diet [19-21]. The results bear on a recent paper indicating that physicians were more likely to use a carbohydrate-restricted diet (CRD) themselves and recommend a LF diet for their patients [22].

This result should be seen in the context of what might be described as a quandary for most family practitioners – surveys generally show a strong feeling among physicians of the importance of nutritional counseling but a limited ability to provide such counseling [23-25] due to a lack of training, limitations of time or adequate reimbursement as well as low confidence in their own ability to advise or patients' ability to comply. In addition, there is a palpable negative response of the media and a documented bias in the nutritional community to low carbohydrate diets [19-21]. A recent search on "Atkins" at the website of American Academy of Family Physicians [26], for example, produced only one hit pointing to their page of "Fad diets" which includes just about any popular diets – in other words, not just low carbohydrate diets but any diet that is selected by individual patients is a fad. This is consistent with the recent release of the No-Fad Diet by the American Heart Association [27] which, while it does not mention any diet by name in the book, lists low carbohydrate diets and the grapefruit diet – the generic fad diet; does anybody know what the grapefruit diet is? – on the dust jacket. Although again, the ALCF is a pre-selected group and we do not know how representative of the American population they are, it is our opinion that 80,000 people is a large number for official agencies to dismiss in such a cavalier fashion. Again, individual practitioners appear to be open-minded and able to consider individual success important.

### Sources of information

#### Questions 49.-56

asked about where people obtained information. The results are shown in Table 2. and are as expected for a group following a strategy that is generally considered outside of the mainstream of recommended medical and nutritional practice, that is, they did not put much stock in official sources. Half of the respondents said they felt that they relied on original scientific publications. On the question of access to the scientific literature (**Q**. 57), they had this opinion:

**Table 2:** Sources of Information

**Source**	**Not important**	**Somewhat important**	**Very important**
**Popular books**	24	40	37
**TV or other media**	71	24	5
**Manufacturers websites**	35	40	25
**Private health associations**	74	20	7
**Online support forums**	10	29	61
**Government websites/publications**	76	19	4
			
**Original scientific publications**	50	34	16

Answers to questions 49–56 [see Additional file 1].

Generally inadequate access (important articles not accessible): 360 (20.37%)

Adequate (was able to see most articles I wanted): 1085 (61.40%)

More than adequate (could not read everything that was available): 322 (18.22%

Posts on the forum reinforce the notion that not only are official recommendations not a source of information, they are in fact viewed with suspicion.

The following post is not uncommon:

The "health experts" are telling kids and parents the wrong foods to eat. Until we start beating the "health experts" the kids won't get any better. If health care costs are soaring and type 2 diabetes and its complications, as are most of these expenses – why are we not putting a "sin" tax on high glycemic foods to cut consumption and help pay for these cost? Beat the "health experts" – not the kids!

From the same member:

I'm not saying that it is all ignorance or all apathy – but there is a lot of ignorance out there – because of what the "health experts" are telling the kids and parents what is healthy. At the expense of repeating myself for the umpteenth time here is what the "health experts" are saying is healthy: .{Wake County Public School System, #873}

Until I researched it three years ago – I thought the most important thing was low fat. So I was eating the hell out of low fat products and my health continued to get worse. ...First link is a school menu and has the comment: "This is why kids are fat. Note in the left hand column the healthy foods are animal crackers, pretzels, cake, cookies, ice cream, pudding, milkshake, juicy juice (sugar water). Look at all the healthy options for breakfast! Can it get any worse?"

### What do people eat on a low carbohydrate diet?

Carbohydrate restriction is not well defined. Anything less than 50 % of the diet is considered by some to be a low carbohydrate diet. From this perspective, the American public at large was on a low carbohydrate diet before the obesity epidemic when compared to the diet during the epidemic and certainly compared to the 55–70 % recommended by health agencies. The problem is compounded by the fact that CRD are frequently hypocaloric by design or due to spontaneous reduction in eating, suggesting that percentages may be misleading. Of greater importance is probably the question of what replaces carbohydrate. Several questions in the survey bear on this. First, a fairly general question (**Q. 4**.) asked for "factors that were important in your low carb diet." Responses are shown in Table 3:

**Table 3:** Responses to question 4. of the survey: which of these factors were important in your weight loss plan? (check all that apply)

**Rank**	**Factor**	**Number**	**% total responders**	**Number 30+/1 yr**	**% 30+/1 yr responders**
**1**	Avoiding sugar	2942	94	1368	96
**2**	Avoiding starch	2629	84	1242	87
**3**	Drinking water	2400	77	1088	76
**4**	Eating vegetables	1969	63	944	66
**5**	Exercise	1879	60	823	58
**6**	Increasing protein	1680	54	773	54
**7**	Avoiding soft-drinks	1271	41	566	40
**8**	Increasing fat	784	25	409	29
**9**	Eating fruits	494	16	205	14
**10**	Decreasing fat	374	12	129	9

Data for all respondents as of January 24, 2006. Note that percentages are for total respondents. Data in Question 4 [see Additional file 1] gives percentage for distribution as of September 18, 2005.

The importance of drinking water was somewhat surprising. Although a consistent recommendation of low carbohydrate and traditional diets alike, to our knowledge, it is not based on any real scientific evidence. It is possible that the water replaced sweetened soft drinks which would, of course, have had a significant impact on weight loss, but we did not ask this question directly.

The importance of vegetables was consistent with narrative posts on the forum, anecdotal information and was further reinforced by our more detailed study of food consumption (**Q. 6.-18.**) which asked about foods that were substituted for those carbohydrates that were removed from the diet. Of total respondents, 53 % (1566) said that they had increased their consumption of Lettuce/Salad Greens greatly (at least double usual consumption) (**Q. 16.**) and 32 % (953) said that they had increased consumption slightly. Results for the 30+1 yr group were similar but more pronounced for the greatly increased category compared to slightly increased (58 % and 28 %, respectively). Results for consumption of green vegetables showed that increased slightly or greatly for the entire group was 79 and 83 % for the 30+1 yr group.

**Figure 2:**
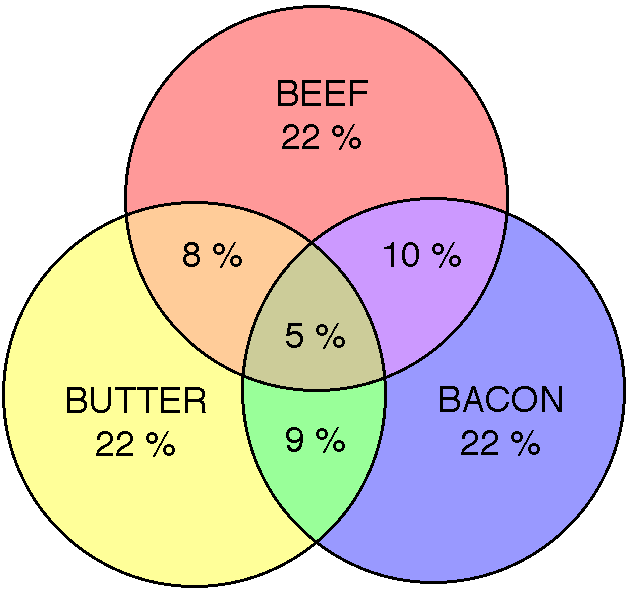
Venn diagram (not to scale) of greatly increased consumption of indicated foods.

These results are in distinction to responses to what might be called the three B's mentioned by critics of low carbohydrate diets: beef, butter and bacon. Although most people in the survey increased these at least slightly, Figure 2 shows that in the category of increased greatly, only 22 % increased one of these foods, 10 % increased two of these foods, and only 5 % had large increases in all. Significantly, these percentages are about the same for the 30+1 yr group.

The actual behavior with respect to vegetable consumption (**Q**. 26.) was as anticipated from assessment of posts on the forum: 40 % (1260) of respondents checked the choice "I don't count carbs in non-starchy vegetables and simply eat all I want." We are grateful to a referee for pointing out that since many low carbohydrate diets specify grams of carbohydrates, these dieters are either not truly counting carbohydrates or are exceeding their targets.

### Food consumption. Calories and portion size

Strategies for weight loss based on carbohydrate restriction tend to downplay the importance of conscious monitoring of caloric intake or portion size. It is generally observed in practice that there is a spontaneous reduction in caloric intake usually attributed to the satiating effect of protein. In our view an additional factor may be relief from the highly reinforcing effect of carbohydrate. In any case, in combination with the psychological benefit of freedom from constant monitoring of calories, non-cognitive regulation of total food intake is one of the major advantages to low carbohydrate approaches and is now appreciated by nutritional experts [28]. Similarly, portion size is generally not specified in low carb diets and macronutrient composition appears to be sufficient to regulate total intake. Our own undocumented guess is that a patient following a low carbohydrate diet who regularly eats a large steak, large potato and large portion of vegetables, rather than reducing the size of each as in official recommendations will simply remove the potato (and is unlikely to add another steak). In that sense, a low carbohydrate diet may more closely resemble behaviorally the habitual American diet than a low fat diet.

In addition to spontaneous caloric reduction, numerous reports in the literature point to an advantage of increased energy inefficiency with carbohydrate restriction leading to more weight lost per calorie consumed (Reviews: [29-32]). Popularly known as metabolic advantage, the effect is more controversial and is not universally accepted even in the face of experimental evidence. The proposed mechanisms for a shift in metabolic efficiency are the increased costs of processing protein for gluconeogenesis, increased substrate cycling or the accumulated kinetic effects of increasing lipolysis over TAG synthesis. The effect is not always seen, however, and little is known about what particular behaviors are required to bring it about.

This question was addressed in the survey by asking respondents about their perception of how total amount of food consumed had changed since being on a low carbohydrate diet (**Q. 31.**). Half of respondents (49 %, 1524) felt that they consumed fewer calories than before the diet, a value that was the same for the 30+1 yr group (49 %, 691). Of the remainder, 30 % said that the total calories are about the same, and 21%, that they felt as though they consumed more calories than before the diet. This result was the same for the group that had lost 30 lbs or more for a year. Thus, to the extent that this is an accurate assessment of their true intake, the results from the people who ate the same amount would support the notion of energy inefficiency since a eucaloric diet with substantial weight loss is *effectively *hypocaloric. Question **30 **asked about portion size and 44 % felt that they ate about the same portion size although 12 % thought that they had eaten somewhat larger portions than before the diet. The accuracy of these perceptions is unknown. It is generally observed that dieters under-report their consumption, although a number of researchers have claimed that dieters on low carbohydrate diets over-report intake but this has not been experimentally demonstrated [1,2]. Narrative reports (**Q. 59**) indicate that the respondents to the survey have consistently been monitoring food throughout their lives and the results are likely to be qualitatively accurate. In any case, half of the respondents at least had the perception that they had either increased their food intake or maintained the amount of food in the face of substantial weight loss which is presumably a motivating factor for compliance. This perception is not generally considered a feature of low fat diets.

**Figure 3:**
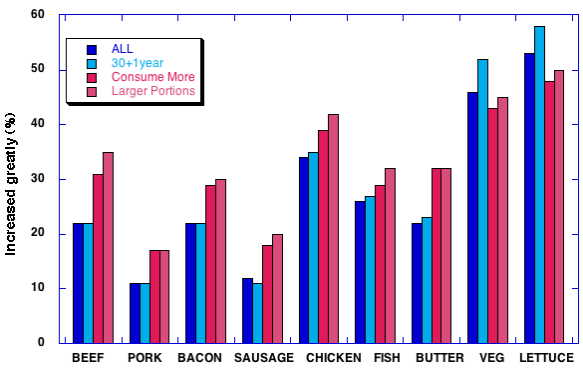
Percentage of respondents indicating greatly increased consumption. The populations were all respondents to the survey, those who had lost 30 lbs and kept the weight off for one year or more (**Q. 2**), those who felt they had consumed more calories that before their diet (**Q. 31**) and those who felt they ate larger portions (last two choices in **Q.30**)

We filtered answers on **Question 31**, isolating the 21 % of respondents who felt that they "consume more calories than before the diet." The food consumed by this subgroup was different than the group at large or the 30+1 yr group. As shown in Figure 3, a higher percentage thought that they had significantly increased (more than double) their intake of meat, fish and butter, the largest effect being seen in the larger percentage who had increases of beef (32 % compared to 21 %). This subgroup was also somewhat less likely to have increased their consumption of vegetables although if they actually eat more food may have consumed the same absolute amount as the group at large. Similar results were seen when we filtered on the combined subgroups that either ate somewhat larger portions or much larger portions (**Q. 31**).

In summary, of the food categories that were perceived as greatly increased by low carbohydrate dieters, the greatest percentage of people had a diet characterized by increased vegetables and salad greens but the subgroup that felt they had increased the total amount of food (possible evidence of decreased energy efficiency) had the largest percentage of people who had increased consumption of meat of all types and butter.

### Relation to CCARBS study

To our knowledge the only systematic internet study of the behavior of dieters using strategies based on carbohydrate restriction is the CCARBS study, an Internet-based prospective study collecting data annually on 2,357 participants [33]. The self-chosen cohort is similar to the group studied here: predominantly female (88%), middle-aged (48 ± 11 yrs) and significantly overweight or obese (BMI at baseline 33.05 ± 8.36 kg/m^2^). A dietary history questionnaire was administered at baseline and at annual time points. At the 1 yr time point, those who had lost weight consumed fewer calories and less carbohydrate but more protein. Like the low-carbers group, the CCARBS population who lost weight consumed more non-starchy vegetables but fewer servings of grain. The group had favorable opinions on low carbohydrate diets and 90.3 % stated that they were less hungry than on a conventional low calorie diet.

### Narrative responses and medical problems

An unintended benefit of the survey is access to attitudes of the subgroup. The last question in the survey "Please feel free to use this space to add any additional comments" was quite general and it was anticipated that it would elicit comments on the questionnaire itself. In fact, almost 1000 responses indicating personal reactions to low carbohydrate dieting were received. We think this provides a remarkable insight into actual behavior of low carbohydrate dieters. Similarly, an open-ended question asked if any improvements in health conditions were noted also. The most common comment was that the respondents felt they "had more energy." This is common from anecdotal information and posts on the forum as well. Due, at least in part, to weight loss, it may also reflect relief from the documented soporific effects of high carbohydrate diets. Table 4 tabulates some of the many health conditions that were reported to have improved. Some, like PCOS, are known to be associated with high insulin and the effectiveness of carbohydrate restriction is documented [34,35]. Others may be coincidental or a reflection of general improved health or weight loss. These will be discussed in detail in a future publication.

**Table 4:** Examples of Reported Changes in Health in answer to question

**Examples of Reported Changes in Health**
"I no longer have diabetes, high blood pressure, sleep apnea, joint pain, back pain and loss of energy.''
"I started low carbing for diabetes. My 3 month blood sugar was 8.9 when diagnosed. It is now 5.4. My doctor is thrilled with my diabetes control and as a side benefit, I lost all that weight! "
"I'm controlling my diabetes without meds or injecting insulin (with an a1c below 5), my lipid profile has improved, I've lost weight, I've gained both strength and endurance, and I've been able to discontinue one of my blood pressure meds.''
"I have much more energy, fewer colds or other health problems. I was able to go completely off oral diabetes medication.''
"All health issues have improved. I am now exercising, sleeping better, asthma is improved, diabetes is constantly under control, high blood pressure maintaining normal levels, no longer constantly depressed, not constantly fatigued.''
"My blood sugar is controlled, cholesterol lowered, acid reflux gone, edema gone, skin clear and healthy. I have chronic low blood pressure and used to have dizzy spells if I stood up too quickly, but those are gone since I've been low carbing."
"For years I had not menstruated regularly at all, but upon starting the diet I have a completely normal menstrual cycle.''
"I have not had any allergic asthma episodes since starting low carb. I have more energy and generally feel better when I stick to low carb eating.''
"Cutting out sugar cleared up a constant condition of plugged sinuses.''
"I have Crohns Colitis. I have noticed a tremendous improvement in that condition since I have cut out sugar in all forms. I have much more energy and I just feel better in general.''

Selection from answers to Question 41.

### The Atkins diet for the family practitioner

Low carbohydrate dieting may be driven by personal recommendations. The family practitioner is likely to be approached by a patient whose acquaintance had good success with one or another of these diets. The results described here present a view of a part of the population where CRD has made an extremely positive impact on their life. The variability in the answers to the questionnaire and the narrative responses indicate there is not even a single Atkins diet and that great flexibility is available in making recommendations on these diets . In the popular mind, and in the mind of many professionals, the Atkins diet means the large increases in saturated fat. There is a serious question about whether the importance of saturated fat is exaggerated – its impact is likely to be very different on a carbohydrate-restricted diet than on others [16,36,37] – but in any case, only about 20 % of respondents had the perception of greatly increasing the amount of foods with high saturated fat. The major change in the intake for most respondents was an increase in non-starchy vegetable consumption and the average diet that emerges from the ALCF is a carbohydrate restricted diet that is high in non-starchy vegetables, low in fruit and only slightly higher in meat compared to respondents' baseline. Such an approach does differ from currently favored diets from most official sources. Current recommendations call for increases in fruits and vegetables, a grouping that does not seem to have much nutritional basis: on average, per 100 g, vegetables have fewer calories than fruits, fewer carbohydrates, more antioxidants, more potassium and are more likely to be integral to a meal rather than consumed in addition to a meal.

Turning this around, the family practitioners can offer a strategy that was appealing to people who describe themselves as being on the Atkins diet (and a subset who maintained large weight loss) and which is not particularly iconoclastic. The data suggest that low fat should not be recommended but neither should increases in fat or protein be required.

On the last point, the recent reports of Women's Health Initiative highlight the limitations of a low fat recommendation. After a period of 7–9 years of low fat diets, a large cohort of women on average lost no weight and showed no improvement in risk for CVD or stroke [38,39]. This result was, in fact, anticipated by the original Seven Countries Study [40], the Framingham study [41,42], the Tecumseh study [43] and the Nurses Health Study[44,45]. The continued emphasis on reduction in fat can no longer be considered part of scientific knowledge.

## Summary

The ALCF offers evidence for the family physician that carbohydrate restriction is one of the useful choices for weight loss and general improvement of health. The narrative reports allow access to patient perceptions and may be more useful in evaluating diets than official recommendations. The evidence from the survey suggests physicians who have been presented with patients desire to reduce carbohydrates are, in fact, open-minded on the subject. The negative connotations given by experts to the term "Atkins diet" may not be appropriate and the actual or perceived behavior of people who identify themselves as being on such a diet allow physicians to design a diet that is likely to be efficacious while not appearing iconoclastic. This last is probably the most important lesson that can be learned from the Active Low-Carber Forums.

## Abbreviations

ALCF: Active Low-Carber Forums   

CRD: carbohydrate-restricted diet   

NWR: National Weight Registry   

UCCASS:Unit Command Climate Assessment and Survey System   

## Competing interests

MCV has held a consulting relationship with Atkins Nutritionals, Inc.

## Supplementary Material

Additional File 1 Feinman_Survey_Results. Survey Results from the Active Low Carber Forums Click here for file
