# Supplementary material for: Low carbohydrate diets in family practice: what can we learn from an internet-based support group
Source: Nutr J. 2006 Oct 2;5:26. doi: 10.1186/1475-2891-5-26 (PMC1609174; doi:10.1186/1475-2891-5-26)
Supplement: Additional File 1 — Feinman_Survey_Results. Survey Results from the Active Low Carber Forums [file 1475-2891-5-26-S1.pdf]

Atkins diet and low carb discussion provided free for information only, not as medical advice.

Home Plans Tips Recipes Tools Stories Studies Products

Active Low-Carber Forums

A sugar-free zone

American Red Cross Victims of Hurricane Katrina need your help now...

Introduce yourself

Hello smonitor, our records indicate that you have never posted to our site before! Why not make your first post today by saying hello to our community in our Introductions forum. With so many active, expert and friendly low-carbing members and millions of posts across dozens of specialty forums, Active Low-Carber Forums is a great place to find and give low-carb support!

Survey Results

Welcome, smonitor.
You last visited: Today at 00:47

Survey Results

Results display options

Results for Survey #28: Nutritional Information from an Internet-based Support Group: What can we learn from the Active Low-Carber Forums?
Questions marked with a [*] were required.

Filter On Checked Questions Go [?]

Survey Time Stats
Average Completion Time: 11min 28sec (Min: 0min 0sec, Max: 149min 7sec)
Average Time before Quit: 9min 10sec

Survey Questions

The purpose of the study is to determine the eating patterns, attitudes and general WOE of members of the forum as an example of a group following a low carb lifestyle.

Carbohydrate restriction continues to be of importance as a method for weight reduction and treatment for diseases such as diabetes and cardiovascular disease. Scientific studies, however, are largely restricted to an abstract, experimental setting and there is a lack of information as to what people really do on low carbohydrate diets and how they feel about them. This survey is designed to help provide this information. The purpose is neither to support nor to criticise any diet but only to provide information.

Confidentiality: all information is strictly confidential and will be reported as group data unless individual permission is obtained in advance. In the final publication, posts on the forum may be presented. We will not use these without members' prior permission and no identifying ID will be used.

There are 27 questions in this survey. With subsections, there are a total of 59 multiple choices. The survey will take 5-10 minutes.

1. [*] Have you lost 30 lbs (or more) on a low-carb plan?

Yes	1502	64.77%
No	414	17.85%
I was not 30 lbs above my target weight	346	14.92%
I did not go on low carb for weight loss	57	2.46%
**Total Answers**	**2319**	

2. [*] Have you kept at least 30 lbs off for one year or more?

Yes	1108	47.80%
No	792	34.17%
I was not 30 lbs above my target weight	361	15.57%
I did not go on low carb for weight loss	57	2.46%
**Total Answers**	**2318**	

3. [*] Which low-carb plan did you follow for losing weight?

Atkins	1259	54.29%
Protein Power	74	3.19%
South Beach Diet	76	3.28%
My own variation of Atkins	471	20.31%
My own variation of other published low carb diets	188	8.11%
Other (low carb)	220	9.49%
Other (not low carb)	31	1.34%
**Total Answers**	**2319**	

4. Which of these factors where important in your weight loss plan? (check all that apply)

Avoiding sugar	2205	17.86%
Avoiding soft-drinks	947	7.67%
Avoiding starch	1990	16.12%
Decreasing fat	262	2.12%
Increasing fat	625	5.06%
Increasing protein	1250	10.12%
Exercise	1395	11.30%
Drinking water	1785	14.46%
Eating fruits	372	3.01%
Eating vegetables	1516	12.28%
**Total Answers**	**12347**	

Obviously on a low-carb plan, you reduce carbohydrates. Which of the following have you increased to replace those carbohydrates that you cut out? (Check about the same for overall if you have simply cut out carbs without replacement).

5. Overall

less than usual	- 294	20.03%
about the same	- 630	42.92%
increased slightly	- 427	29.09%
increased greatly (at least double usual consumpion)	- 117	7.97%
**Total Answers**	- **1468**	

6. Beef

less than usual	- 117	5.35%
about the same	- 674	30.83%
increased slightly	- 950	43.46%
increased greatly (at least double usual consumpion)	- 445	20.36%
**Total Answers**	- **2186**	

7. Pork

less than usual	- 202	9.51%
about the same	- 932	43.86%
increased slightly	- 764	35.95%
increased greatly (at least double usual consumpion)	- 227	10.68%
**Total Answers**	- **2125**	

8. Bacon

less than usual	- 277	12.96%
about the same	- 669	31.29%
increased slightly	- 744	34.80%
increased greatly (at least double usual consumpion)	- 448	20.95%
**Total Answers**	- **2138**	

9. Sausages

less than usual	- 403	19.18%
about the same	- 841	40.03%
increased slightly	- 618	29.41%
increased greatly (at least double usual consumpion)	- 239	11.38%
**Total Answers**	- **2101**	

10. Chicken

less than usual	- 45	2.04%
about the same	- 503	22.79%
increased slightly	- 945	42.82%
increased greatly (at least double usual consumpion)	- 714	32.35%
**Total Answers**	- **2207**	

11. Fish

less than usual	- 113	5.22%
about the same	- 710	32.78%
increased slightly	- 812	37.49%
increased greatly (at least double usual consumpion)	- 531	24.52%
**Total Answers**	- **2166**	

12. Olive Oil

less than usual	- 103	4.76%
about the same	- 818	37.78%
increased slightly	- 795	36.72%
increased greatly (at least double usual consumpion)	- 449	20.74%
**Total Answers**	- **2165**	

13. Canola Oil

less than usual	- 645	32.25%
about the same	- 1045	52.25%
increased slightly	- 235	11.75%
increased greatly (at least double usual consumpion)	- 75	3.75%
**Total Answers**	- **2000**	

14. Butter

less than usual	- 226	10.45%
about the same	- 621	28.72%
increased slightly	- 845	39.08%
increased greatly (at least double usual consumpion)	- 470	21.74%
**Total Answers**	- **2162**	

15. Green Vegetables

less than usual	- 73	3.30%
about the same	- 335	15.15%
increased slightly	- 747	33.79%

increased greatly (at least double usual consumpion)	-	1056	47.76%
**Total Answers**	-	**2211**	

16. Lettuce / Salad Greens

less than usual	-	48	2.17%
about the same	-	273	12.34%
increased slightly	-	705	31.87%
increased greatly (at least double usual consumpion)	-	1186	53.62%
**Total Answers**	-	**2212**	

17. Berries

less than usual	-	527	24.52%
about the same	-	575	26.76%
increased slightly	-	649	30.20%
increased greatly (at least double usual consumpion)	-	398	18.52%
**Total Answers**	-	**2149**	

18. Fruits

less than usual	-	1277	60.26%
about the same	-	497	23.45%
increased slightly	-	247	11.66%
increased greatly (at least double usual consumpion)	-	98	4.62%
**Total Answers**	-	**2119**	

19. [*] What is your gender?

Male	-	434	18.71%
Female	-	1886	81.29%
**Total Answers**	-	**2320**	

20. [*] What is your age group?

Under 20	-	29	1.25%
Between 20-29	-	317	13.66%
Between 30-39	-	705	30.39%
Between 40-49	-	713	30.73%
Between 50-59	-	408	17.59%
Between 60-69	-	132	5.69%
70 or over	-	16	0.69%
**Total Answers**	-	**2320**	

21. [*] Have you had blood lipids measured before and after going on a low carb diet?

Yes	-	986	42.35%
No	-	1342	57.65%
**Total Answers**	-	**2328**	

If you had your blood lipids measured, please describe the effect on numbers, if you know:

22. Total cholesterol

Increased	-	125	11.93%
No change	-	262	25.00%
Decreased	-	661	63.07%
**Total Answers**	-	**1048**	

23. LDL

Increased	-	108	11.63%
No change	-	257	27.66%
Decreased	-	564	60.71%
**Total Answers**	-	**929**	

24. HDL

Increased	-	487	52.09%
No change	-	261	27.91%
Decreased	-	187	20.00%
**Total Answers**	-	**935**	

25. Triglycerides

Increased	-	38	3.99%
No change	-	236	24.76%
Decreased	-	679	71.25%
**Total Answers**	-	**953**	

26. Some people on low carb diets say that they don't count the carbs in non-starchy vegetables like broccoli and lettuce, and eat as much as they want of these foods. What is your experience when on the weight loss part of your diet?

I count all carbs as much as I can.	-	677	29.28%
I eat all I want but figure in daily carb count.	-	291	12.59%
I count roughly and limit the amount of vegetables.	-	387	16.74%

I don't count carbs in non-starchy vegetables and simply eat all I want.	-	957	41.39%
**Total Answers**	-	**2312**	

27. Are there any high carbohydrate foods that you consistently crave?

I rarely have any cravings for excluded carbs.	-	460	19.90%
I only have occasional cravings for excluded carbs which I just ignore.	-	532	23.02%
I have occasional cravings but I just eat small amounts of the food I crave.	-	481	20.81%
I have occasional cravings but I try the low carb version of the food (Low-carb candy bars, for example)	-	669	28.95%
I consistently crave some foods that are forbidden and am unable to satisfy this craving.	-	169	7.31%
**Total Answers**	-	**2311**	

28. If there are foods that you consistently crave, which ones are they (check all that apply):

None	-	496	10.71%
Fruit	-	402	8.68%
Potatoes, any style	-	427	9.22%
Pastry	-	329	7.10%
Pasta	-	372	8.03%
French fries	-	331	7.15%
Cookies	-	433	9.35%
Cheese Cake	-	210	4.53%
Candy	-	423	9.13%
Bread	-	648	13.99%
Rice	-	223	4.82%
Other	-	337	7.28%
**Total Answers**	-	**4631**	

29. In staying on a low carbohydrate diet:

I mostly eat the foods I always ate but leave out the carbohydrates.	-	913	39.44%
I eat more of certain foods but mostly already prepared food.	-	113	4.88%
I have learned to prepare new kinds of food.	-	1289	55.68%
**Total Answers**	-	**2315**	

30. How does the portion size or total volume of most of your meals compare to what you ate before your diet?

I have substantially reduced the size of portions I eat.	-	296	12.79%
I generally find I eat somewhat smaller portions than before.	-	663	28.64%
I eat about the same portion size, just different food.	-	1047	45.23%
I generally eat somewhat larger portions than before.	-	265	11.45%
I eat much larger portions.	-	44	1.90%
**Total Answers**	-	**2315**	

31. Overall, in your perception, how has the total amount of food (total calories) that you consume changed since you have been on a low carbohydrate diet.

I feel as though I consume less calories than before the diet.	-	1149	49.68%
I think the total calories are about the same.	-	700	30.26%
I feel as though I consume more calories than before the diet.	-	464	20.06%
**Total Answers**	-	**2313**	

32. [*] Did you consult a physician or other health professional before or during your low carbohydrate diet?

Yes	-	1176	50.49%
No	-	1153	49.51%
**Total Answers**	-	**2329**	

33. If you have consulted a physician or other health professional , how would you describe their support for your low carb plan?

The physician or health professional was supportive of my low carb diet.	-	736	55.17%
They did not have an opinion but were encouraging after seeing results.	-	378	28.34%
They were opposed to my low carb diet and offered an alternative.	-	133	9.97%
They were discouraging even after I showed good results.	-	87	6.52%
**Total Answers**	-	**1334**	

34. When, approximately, did you start low carbing?

Total Answers - **2086**

[View Answers]

35. How much did you want to lose? (in lbs)

0 - 9 lbs.	-	79	3.59%
10-29 lbs	-	418	19.01%
30-49 lbs.	-	509	23.15%
50-99 lbs	-	695	31.61%
100 lbs or more	-	498	22.65%
**Total Answers**	-	**2199**	

36. Have you reached your weight loss goal, or within 10 lbs of your goal?

Yes	-	863	37.36%
No	-	1314	56.88%
Not Applicable	-	133	5.76%
**Total Answers**	-	**2310**	

37. If yes, what is the approximate date?

Total Answers - **794**

[View Answers]

38. Were you able to maintain at least 30 lbs of the weight you did lose for a year or more?

Yes	-	1270	67.95%
No	-	599	32.05%
**Total Answers**	-	**1869**	

39. If you have recorded your weight loss, please list the approximate dates and the recorded weight next to them. Fell free to cut and paste in any form you might have or use the following format, with approximate months or quarters:

Start: June 2002 - 260 lbs
July 2002 -240 lbs
Oct 2002 220 lbs
.....
Reached goal July 2003 - 180 lbs
Current weight: July 2005 - 185 lbs.

Total Answers - **988**

[View Answers]

40. How would you describe your satisfaction with low-carb plans?

Best plan I ever tried	-	1389	60.58%
Satisfied	-	503	21.94%
It's o.k.	-	223	9.73%
It was hard	-	121	5.28%
It didn't work	-	33	1.44%
I hate it	-	24	1.05%
**Total Answers**	-	**2293**	

41. Any noticeable changes to your health? If so, please specify

Total Answers - **1619**

[View Answers]

Rate the following likes and dislikes about the impact of following a low carb plan. Select "no change" if you have not noticed any changes:

42. Variety of food

Greatly improved	-	569	24.85%
Slightly improved	-	596	26.03%
No change	-	402	17.55%
Slightly worse	-	614	26.81%
Much worse	-	109	4.76%
**Total Answers**	-	**2290**	

43. The way I feel

Greatly improved	-	1654	71.85%
Slightly improved	-	475	20.63%
No change	-	117	5.08%
Slightly worse	-	41	1.78%
Much worse	-	15	0.65%
**Total Answers**	-	**2302**	

44. My sleep pattern

Greatly improved	-	735	31.94%
Slightly improved	-	669	29.07%
No change	-	806	35.03%
Slightly worse	-	73	3.17%
Much worse	-	18	0.78%
**Total Answers**	-	**2301**	

45. Clarity of thinking

Greatly improved	-	694	30.23%
Slightly improved	-	685	29.83%
No change	-	845	36.80%
Slightly worse	-	61	2.66%
Much worse	-	11	0.48%
**Total Answers**	-	**2296**	

46. Mood

Greatly improved	-	863	37.57%
Slightly improved	-	775	33.74%

No change	-	559	24.34%
Slightly worse	-	81	3.53%
Much worse	-	19	0.83%
**Total Answers**	-	**2297**	

47. Feeling of Hunger

Greatly improved	-	1140	49.57%
Slightly improved	-	658	28.61%
No change	-	354	15.39%
Slightly worse	-	132	5.74%
Much worse	-	16	0.70%
**Total Answers**	-	**2300**	

48. Energy

Greatly improved	-	1260	54.69%
Slightly improved	-	706	30.64%
No change	-	257	11.15%
Slightly worse	-	65	2.82%
Much worse	-	16	0.69%
**Total Answers**	-	**2304**	

To what extent were any of the following sources important to you in selecting a diet and in understanding nutrition in relation to your diet

49. popular books

not important	-	534	23.39%
somewhat important	-	894	39.16%
very important	-	855	37.45%
**Total Answers**	-	**2283**	

50. TV or other media

not important	-	1600	71.52%
somewhat important	-	536	23.96%
very important	-	101	4.51%
**Total Answers**	-	**2237**	

51. Websites of manufacturers (like Atkins)

not important	-	802	35.52%
somewhat important	-	895	39.64%
very important	-	561	24.84%
**Total Answers**	-	**2258**	

52. Private health associations (like American Diabetese Association)

not important	-	1674	75.24%
somewhat important	-	412	18.52%
very important	-	139	6.25%
**Total Answers**	-	**2225**	

53. Online support forums (like this site)

not important	-	236	10.33%
somewhat important	-	650	28.46%
very important	-	1398	61.21%
**Total Answers**	-	**2284**	

54. Government websites or publications (USDA, FDA)

not important	-	1720	77.44%
somewhat important	-	413	18.60%
very important	-	88	3.96%
**Total Answers**	-	**2221**	

55. Original scientific publications.

not important	-	1106	49.44%
somewhat important	-	759	33.93%
very important	-	372	16.63%
**Total Answers**	-	**2237**	

56. If you thought original scientific publications were important, to what extent did you find that you had adequate access (internet, library, etc.)

Generally inadequate access (important articles not accessible)	-	265	20.11%
Adequate (was able to see most articles I wanted)	-	819	62.14%
More than adequate (could not read everything that was available).	-	234	17.75%
**Total Answers**	-	**1318**	

57. Are there any foods that are nominally low carb or moderate carb (ingredient labels, etc.) have the effect of stopping or curtailing weight loss for you, even at recommended levels?

Cheese	-	463	22.88%
Peanuts	-	628	31.03%
Other nuts	-	512	25.30%
Sour Cream	-	131	6.47%
Yoghurt	-	290	14.33%
**Total Answers**	-	**2024**	

58. Have you heard of the National Weight Registry and if so, have you tried to register?

No, I never heard of it.	-	1997	86.60%
Yes, but I did not meet the criteria.	-	72	3.12%
Yes, but have not attempted to register	-	213	9.24%
Yes, I met their criteria and tried to register but never heard from them.	-	18	0.78%
Yes, I am a member.	-	6	0.26%
**Total Answers**	-	**2306**	

59. Please feel free to use this space to add any additional comments.

Total Answers - **746**

[View Answers]

Copyright © 2000-2005 Active Low-Carber Forums @ forum.lowcarber.org
Powered by: vBulletin, Copyright ©2000 - 2005, Jelsoft Enterprises Ltd.

Powered by UCCASS 1.8. Copyright © 2004 John W. Holmes, All Rights Reserved
